# Cycling matters: Sex hormone regulation of vascular potassium channels

**DOI:** 10.1080/19336950.2023.2217637

**Published:** 2023-05-27

**Authors:** Samuel N Baldwin, Thomas A Jepps, Iain A Greenwood

**Affiliations:** a Vascular Biology Group, Department of Biomedical Sciences, University of Copenhagen, Copenhagen, Denmark; b Vascular Biology Research Centre, Institute of Molecular and Clinical Sciences, St George’s University of London, London, UK

**Keywords:** ion channels, vascular biology, estrus cycle, estrogens, progesterone, testosterone

## Abstract

Sex hormones and the reproductive cycle (estrus in rodents and menstrual in humans) have a known impact on arterial function. In spite of this, sex hormones and the estrus/menstrual cycle are often neglected experimental factors in vascular basic preclinical scientific research. Recent research by our own laboratory indicates that cyclical changes in serum concentrations of sex -hormones across the rat estrus cycle, primarily estradiol, have significant consequences for the subcellular trafficking and function of K_V_7. Vascular potassium channels, including K_V_7, are essential components of vascular reactivity. Our study represents a small part of a growing field of literature aimed at determining the role of sex hormones in regulating arterial ion channel function. This review covers key findings describing the current understanding of sex hormone regulation of vascular potassium channels, with a focus on K_V_7 channels. Further, we highlight areas of research where the estrus cycle should be considered in future studies to determine the consequences of physiological oscillations in concentrations of sex hormones on vascular potassium channel function.

## Introduction

Cyclical changes in sex hormones driven by the human or rodent reproductive cycle coincide with changes in vascular function [1]. To date, research regarding the impact of sex hormones on vascular function has focused on sex-hormone-mediated changes in the production of endothelial-derived relaxing factors such as nitric oxide (NO), prostaglandin I_2_ (PGI_2_), or endothelial-derived hyperpolarization (EDH) [2–5]. These pathways depend on the activation of vascular smooth muscle cell (VSMC) potassium (K^+^) channels to elicit their effect. For example, large conductance calcium-activated potassium channels (BK_Ca_), voltage-gated potassium channels (K_V_), and inwardly rectifying potassium channels are activated in response to NO [6,7], PGI_2_ [8–12], and EDH [13,14], respectively. Yet, comparably little is known about the influence of sex hormones on vascular smooth muscle K^+^ channels. Recently, Baldwin *et al.* 2022 and 2023 showed the functional impact of arterial KCNQ-encoded voltage-dependent potassium channels (termed Kv7s) changed considerably across the rat estrus cycle [12,15]. These works are in the vanguard of research identifying estrus-cycle-dependent oscillations in smooth muscle functionality outside of the sex-dependent differences in arterial responsiveness conventionally reported [16]. Alterations in smooth muscle ion channel activity driven by sex hormone oscillations have potential implications in disease manifestations and pharmacological treatments, highlighting why estrus “cycling” matters. This review provides an overview of this nascent and emerging field of research and draws comparisons with known work in other cell types. It focuses primarily on Kv7 channels, but also includes aspects on how sex and sex hormones regulate other K^+^ channels.

## The estrus cycle

Sex hormones, principally estrogens, progesterone, and androgens, fluctuate throughout the course of the estrus (rodent) and menstrual (human) cycles. A direct comparison between rodent and human cycles is difficult as the rodent estrus cycle last only 4–5 days and is split into four stages (proestrus, estrus, metestrus and diestrus); whereas the human cycle lasts roughly 28 days and has three phases (follicular, ovulation and luteal), followed by menses. Deducing the impact of sex hormones on vascular reactivity throughout the menstrual cycle is challenging, not least because of additional genetic and environmental factors that will also drive significant changes in human vascular reactivity. Thus, there is a paucity of human data describing arterial function changes throughout the menstrual cycle. As such, this review will focus primarily on the rodent estrus cycle, which is described comprehensively by Nilsson *et al.* (2015) [17]. A brief overview of each hormone and their vascular receptors will be given here.

## Estrogen

The bioactive form of estrogen, 17-β estradiol (E2), is synthesized in the theca and granulosa cells of the ovaries, the adrenal glands and locally within the vasculature by aromatase enzymes. E2 produces both genomic and fast acting non-genomic vascular effects through nuclear- and membrane-bound estrogen receptors (ERs). ERα/ERβ, which are canonically considered cytoplasmic nuclear receptors, dimerize to form homo/hetromers upon binding E2, which translocate to the nucleus. Active nuclear ERs bind estrogen response elements within the genome and regulate transcription [18,19]. E2 also exerts rapid non-genomic effects that derive from membrane bound ERα/β [20], which evoke a myriad of intracellular signaling cascades attributed to mitogen activated protein kinase (MAPK)/extracellular signal- regulated kinases (ERK), serine and threonine kinases, phosphoinositide 3-kinase (PI3K), and cAMP [21–24]. ERα/ERβ are ubiquitously expressed across the vasculature [19,25]. A G protein-coupled estrogen receptor (GPER1), designated GPR30 initially, is also expressed in the membrane of the sarcoplasmic reticulum, the golgi apparatus and the plasma membrane of VSMCs and endothelial cells (ECs) [19]. GPER1 couples to a complex array of signaling cascades via G_αs,i,q/ll_ and is further complicated via receptor cross talk with the mineralocorticoid and epidermal growth factor receptors [26,27].

## Progesterone

Progesterone is a steroidal hormone produced within the adrenal cortex, gonads and the corpus luteum of the pregnant rat. Its receptors have been found within both ECs and VSMCs of rats, mice and humans [28]. Compared to E2, the literature on progesterone within the vasculature is sparse, though it can be broken down into nuclear and non-nuclear signaling. Progesterone binds two distinct cytoplasmic nuclear receptors termed progesterone receptor (PR) A/B, which are derived from a single gene. Upon binding progesterone, the receptors dimerize, forming homo/heteromers, which translocate to the nucleus and bind progesterone response elements that subsequently regulate gene transcription [29,30]. Moreover, a novel class of membrane-bound progesterone receptor (mPR) has been discovered and includes three subtypes, α, β and γ. The activation of mPR is associated with G_αi_ and subsequent decrease in cAMP, as well as the activation of MAPKs, ERK1/2, and c-Jun N-terminal kinase (JNK1/2) [31–33].

## Testosterone

Testosterone, and its more potent analogue di-hydro testosterone, are produced within the gonads and adrenal glands of both sexes. Like E2 and progesterone, testosterone activates both nuclear- and membrane-bound androgen receptors (ARs). Classic ARs include two isoforms, A/B, which are cytosolic nuclear transcription factors that dimerize and translocate to the nucleus where they bind androgen response elements, mediating a change in gene transcription [24]. Recently, a novel class of testosterone-activated G protein-coupled receptor was discovered [34,35], in addition to membrane-associated nuclear ARs. Upon binding testosterone, activated membrane-associated nuclear, and membrane-bound G protein-coupled ARs undergo a conformational change in shape, and can mediated rapid non-genomic effects associated with the activation of a protein kinase C (PKC), protein kinase A (PKA), MAPK, cAMP, ERK, and protein kinase G (PKG) [24,34,35]. Testosterone can also be converted to E2 locally within the vasculature by aromatase activity, which complicates delineation between vascular testosterone and E2 signaling.

## K^+^ channels

K^+^ channels are a fundamental factor in the generation of VSMC resting membrane potential, which is roughly −70 to −55 mV [36]. As K^+^ channels open, the cell becomes permeable to K^+^ ions, which move down their electrochemical gradient, exiting the cell, thus moving VSMC membrane potential (*V*_m_) toward the equilibrium potential for K^+^ (E_*k*_). As E_*k*_ within VSMC is roughly −90 mV, the cell membrane becomes hyperpolarized. Despite the known role for K^+^ channels in VSMC reactivity within the male, their role within the female vasculature and how their activity is affected by hormonal fluctuations is poorly understood. K^+^ channels are principally comprised of homo or heterotetrameric configurations of α-subunits that exist within the membrane in associated with β-auxiliary subunits. β-auxiliary subunits regulate the biophysical, physiological and pharmacological properties of the channel. Both the α- and β-subunits are candidate targets for sex hormone regulation of ion channel function, and will be discussed below. See Table 1 for a summary of the effect of sex hormones on vascular and non-vascular K^+^ channels.

**Table 1.** The effect of sex hormones on vascular and non-vascular K^+^ channels. *= hormone mediator speculated.

		Channel function/expression								
		Upregulated			Downregulated			No effect		
Ion channel	Hormone	Subtype	Model	Ref	Subtype	Model	Ref	Subtype	Model	Ref
*K*_*V*_*7*	Estrogen				K_V_7.1	*Xenopus* Oocytes	[37]			
						HT29cl.19A	[38]			
						Rat: Distal colic crypt cells	[38–40]			
					K_V_7.1: KCNE1	Mouse/Guinea pig: Ventricular myocytes	[41]			
					K_V_7.1: KCNE3	Chinese Hamster Ovarian cells	[40]			
					K_V_7.4	Rat mesenteric/renal VSMC	[12,15]			
	Progesterone	K_V_7.1/ K_V_7.5/ Kcne1	Murine uterus*	[42]				K_V_7.2–5	Rat mesenteric artery	[12]
	Testosterone	K_V_7.1	Rat cardiac myocyte	[43]						
		KCNE4	Mouse cardiac myocyte	[44]						
*K*_*V*_*1*	Estrogen				K_V_1.5	Murine ventricular myocytes	[41,45]			
	Progesterone	K_V_1.5	H9C2 cells	[46]	K_V_1.1–6	Murine ventricular myocytes	[46]			
	Testosterone							K_V_1.5	Murine ventricular myocytes	[47,48]
								K_V_1.5	Rat aorta	[49]
*K*_*V*_*2*	Estrogen				K_V_2.1	Human osteoblast-like MG63 cells	[50]			
						Cultured rat hippocampal neurons	[51]			
						Guinea pig heat	[41]			
					K_V_2.1/ 2.2	Murine Β-pancreatic cells	[52]			
					K_V_2.2	Cultured rat cerebellar granule	[53]			
						HEK-293 cells	[53]			
	Progesterone	K_V_2.1	H9C2 cells	[46]				K_V_2.1	HEK293 cells	[54]
*K*_*V*_*11.1*	Estrogen	K_V_11.1	Murine ventricular myocytes	[55]	K_V_11.1	Guinea pig heat	[41,56]			
			Human ECG	[57]		HEK293B	[56,58]			
			HEK293B	[57]		Computational	[59]			
	Progesterone	K_V_11.1	Guinea pig ventricular myocytes	[60]	K_V_11.1	Rat neonatal cardiac myocytes	[61]			
			Computational	[60]		Murine uterus*	[62]			
					K_V_11.1: KCNE2	Human uterus*	[63]			
	Testosterone	K_V_11.1	HEK293B	[64]						
			Rabbit ventricular myocytes	[65]						
*BK*_*Ca*_	Estrogen	BK_Ca_1.1:K_Ca_β1	Rat cerebral arteries	[66]	BK_Ca_1.1	HUV-EC-C	[67]			
		BK_Ca_1.1	Human coronary arteries	[68,69]						
			Porcine coronary arteries	[70]						
			Cultured human coronary/aortic VSMCs	[71]						
			Ovine uterus arteries	[72–74]						
	Progesterone				BK_Ca_1.1	Xenopus oocytes	[75]	BK_Ca_1.1	Ovine uterus arteries	[72]
	Testosterone	BK_Ca_1.1	Porcine coronary arteries	[76,77]						
			Rat mesenteric arteries	[78]						
*K*_*ATP*_	Estrogen	K_ATP_: SUR1, mKATP	Murine myocardium	[79]	K_ATP_	Murine β-pancreatic cells	[80–82]	K_ATP_	Human coronary artery	[83]
		K_ATP_: SUR2A	H9c2	[84]						
		K_ATP_: SUR1	Rat brain cortices	[85]						
		K_ATP_	Murine gonadotrophin releasing neurons	[86,87]						
			Human myocardium	[83]						
	Testosterone	K_ATP_	Canine coronary artery	[88]				K_ATP_	Rat aorta	[89]
			Rat aorta	[90]						
			Human corporal artery	[91]						

## K_V_7 channels

K_V_7 channels are a K_V_ channel subfamily encoded for by the genes *KCNQ1–5*, which give rise to distinct α-subunit proteins, termed K_V_7.1–7.5, respectively. K_V_7 channels activate slowly upon depolarization with a relatively negative threshold compared to other K_V_ channels (~-40 mV) and exhibit little inactivation. Every human or rodent vascular bed studied expresses K_V_7 channel transcripts, with *Kcnq1, Kcnq4*, and *Kcnq5* being the principally expressed genes [92,93]. There is comparably little contribution from *Kcnq3* and rarely expression of *Kcnq2*. K_V_7 channel stoichiometry varies between systems. Vascular K_V_7.1 is commonly thought of as exclusively homotetrameric [94], although Oliveras *et al.* (2014) demonstrated a K_V_7.1/7.5 heteromer within coronary artery VSMCs from rats [95]. Vascular K_V_7.4 readily forms heterotetrameric channels with Kv7.5, but are also found as homotetrameric channels, whereas K_V_7.5 exists predominantly with K_V_7.4 in native VSMCs [96,97]. Within rat [98] and murine [99] mesenteric VSMCs, K_V_7.4 and Kv7.4/7.5 channels exist in close proximity with the KCNE4 β-auxiliary subunit protein, which positively regulates channel trafficking and biophysical/pharmacological properties [99].

K_V_7 channels regulate arterial smooth muscle contractility and are a key component of receptor-mediated vasodilations in arteries from male animals. Application of the pan-K_V_7 channel blockers, linopirdine or XE991, depolarizes rat *V*_m_ [100,101] and mediates a rise in baseline tension in both human and rodent arteries [92,102–105]. In contrast, K_V_7.1-specific blockers like HMR-1556 or chromanol 293B do not effect baseline tension in rat aorta, mesenteric nor intrapulmonary arteries [106]. Functional vascular K_V_7 channel activity is further evidenced by relaxation of pre-contracted arterial tone in response to several K_V_7.2–7.5 channel activators (e.g. retigabine, acrylamide S-1, ML213) across a number of rodent arteries [93,105,107,108]. Similarly, activators of K_V_7.1, including (R)-L3, Mefenamic acid and ML277 [106,108], are effective and reversible relaxants of contracted arteries. These data suggest that K_V_7.4/K_V_7.5 are regulators of resting arterial smooth muscle *V*_m_ due to their low activation threshold and that their activity hyperpolarizes the membrane potential reducing the open probability of voltage-gated calcium channels, thereby negating Ca^2+^ influx. Whereas K_V_7.1 channels do not contribute to resting *V*_m_, but are functionally expressed.

Pan-K_V_7 blockers and molecular knockdown of K_V_7.4/K_V_7.5 channels also impair receptor-mediated vasorelaxation generated by β-adrenoreceptor, adenosine, calcitonin-gene related peptide, and prostacyclin (IP) receptor agonists (cAMP-linked [12,96,104,109,110]), as well as atrial natriuretic peptide and nitric oxide-mediated responses (cGMP-linked [111–113]) in many arteries from male rats. Interestingly, β-adrenoreceptor mediated relaxation differentially couples to K_V_7 in a vascular bed specific manner, via PKA in renal arteries, and a novel alternate signaling cascade mediated by exchange protein activated by cAMP (EPAC) in mesenteric arteries [109]. K_V_7.1-specific blockers do not affect an array of receptor-mediated vasorelaxations, although Baldwin et al. (2022) revealed that relaxations of rat mesenteric artery induced by activation of IP receptors, either with iloprost or MRE-269, were attenuated by HMR1556 to the same degree as the pan-Kv7 blocker linopirdine.

A growing body of literature indicates sexual dimorphisms in vascular K_V_7 physiology. Abbott and Jepps (2015) observed that mesenteric arteries from male *Kcne4-/-* knockout mice showed an increased sensitivity to α-1 adrenoreceptor agonist methoxamine and decreased sensitivity to K_V_7.2–7.5 activator ML213, which was not observed in females [98]. Further, Berg (2018) observed that vascular K_V_7 channel function was preserved in female hypertensive animals when compared to males [114]. As above, Baldwin *et al.* (2022) demonstrated a novel role for K_V_7.1 as the downstream target of IP receptor evoked relaxation in mesenteric arteries from both male and female rats [12]. However, the sensitivity of IP receptor-mediated relaxation to inhibition of K_V_7.1 by pre-incubation of HMR-1556 (10 µmol-L^−1^) was absent in arteries from animals harvested during proestrus or estrus stages of the estrus cycle [12], stages associated with an initial spike in serum concentration of E2 [17]. This correlated with a significant decrease in sensitivity to IP receptor-mediated relaxation when compared to mesenteric arteries from female animals harvested from diestrus/met-estrus, where HMR-1556 still had an effect. These findings were the first demonstration of an estrus cycle-sensitive regulation of arterial K_V_7 function.

Subsequently, Baldwin *et al.* (2023) focused on responsiveness of several K_V_7 channel activators across the estrus cycle of the rat. These experiments revealed that during cycle stages associated with high E2, proestrus/estrus, K_V_7.2–5 channel activator-mediated relaxations were markedly impaired when compared to diestrus/metestrus, where serum E2 was low [15]. Similarly, thromboxane A2 (TXA2) receptor-mediated vasoconstriction was enhanced and β adrenoceptor-mediated relaxation was impaired during proestrus/diestrus when compared to diestrus/metestrus [15]. The sensitivity of these responses to K_V_7 inhibition during proestrus/estrus was also diminished [15]. An impairment of vascular function during proestrus/estrus was associated with a translocation of K_V_7.4 from the plasma membrane. Both K_V_7 function and protein membrane abundance were impaired by application of exogenous E2 and the GPER1 agonist, G1, in arteries from females form low serum E2 stages of the estrus cycle, diestrus/metestrus, but not proestrus/metestrus. These *ex vivo* manipulations were prevented by prior application of the GPER1 antagonist, G36. The findings of Baldwin et al. (2023) were the first to identify a cyclical estrous-regulation of an ion channel in the vasculature and the functional consequences. This work reinforces earlier studies [115–117], indicating that K_V_7.4 channel abundance in the plasma membrane is labile and at the mercy of the arterial environment.

Whilst the impact of sex hormones on vascular Kv7 channels is a nascent field of research, work in other cell types also indicates an acute estrogenic inhibition of K_V_7 function and expression. For example, supraphysiological concentrations of E2 (1 µmol-L^−1^) inhibit K_V_7.1:KCNE1 channel derived currents in an heterologous expression system [37] and the E2 receptor antagonist, tamoxifen, increased K_V_7.1 function and expression in murine and guinea pig ventricular myocytes [41], suggesting a tonic estrogenic suppression of the channel. E2-induced inhibition of K_V_7.1 derived currents in rat distal colic crypt cells occurs in a PKC-δ-dependent manner, independent of the classical estrogen receptors [39], and basolateral K_V_ currents responsible for Cl^−^ secretion are inhibited by E2 via uncoupling of K_V_7.1:KCNE3 [40]. Moreover, in HT29cl.19A cells, E2 abrogated K_V_7.1 membrane expression through PKC-δ-dependent channel endocytosis into early endosomes, which is then recycled to the membrane [38]. Taken as a collective, the aforementioned studies provide strong evidence of estrogenic K_V_7:KCNE channel regulation in a complex process of channel recycling. Within renal arteries from female rats, VSMC KCNE4 membrane abundance did not fluctuate across the estrus cycle [15]. Consequently, the role of KCNE4 in E2 regulation of vascular K_V_7.4 remains to be determined, though a process of E2-mediated vascular K_V_7.4 recycling similar to that described in Rapetti-Mauss *et al.* (2013) is plausible, in light of the rapid recovery of vascular K_V_7.2–5 function between estrus cycle stages [15].

In contrast to E2, short-term supplemental progesterone incubation on ex vivo mesenteric arteries from female rats had no effect on the vasorelaxation elicited by K_V_7.2–7.5 channel activators across the estrus cycle [15]. These data cannot conclusively rule out a role for progesterone on K_V_7 channel function or expression, as K_V_7 channel modulator sensitivity and membrane abundance of K_V_7.4 was enhanced during metestrus/diestrus, where serum progesterone was highest [15]. Similarly, in mouse myometrial tissue, the total copy number for *Kcnq1, Kcnq5* and *Kcne1* increases during metestrus [42]. Future experiments with antiprogestogens, and long-term incubation of arteries (+24hrs) are required to determine the impact of progesterone on vascular K_V_7 channel function.

Within male rodents, testosterone increases the relative expression of *Kcnq1* in cardiomyocytes [43]. This has been postulated to underpin testosterone mediated QT interval shortening [43]. The impact of testosterone signaling on vascular K_V_7 channels has yet to be investigated, however, testosterone upregulates *Kcne4* [44] expression in ventricular myocytes from male mice. *Kcne4* transcript is more abundantly expressed in mesenteric arteries from male mice when compared to females, but Kv7.4 protein expression is lower; however, the responses to ML213 were the same [98]. These data potentially suggest that females have more K_V_7.4 channels but the testosterone-mediated upregulation of *Kcne4* increases the channel function in the males. Though this remains to be determined.

K_V_7 channels are being considered as a novel therapeutic target in the treatment of hypertension and other diseases [118]. Though the gross impact of “estrus cycling” vascular K_V_7 channel function on blood pressure and health has yet to be established, current understanding indicates that the pharmacology required to target the channel conventionally, through activators for example, would change dramatically across the menstrual cycle, thereby excluding these channels as convenient targets in pre-menopausal women.

## K_V_1 channels

Several studies have found transcript and protein expression of different K_V_1 channel subtypes across various vascular beds from multiple animal models, with Kv1.5 predominating [119–126]. K_V_1.5 channels are implicated in regulating myogenic tone [124,125] and receptor-mediated contraction [127] and relaxation [128–132]. Interestingly, K_V_1.3 has been implicated in the phenotypic switch to contractile from proliferative VSMCs via PLCγ, independent of K^+^ influx [133,134], prospectively through their c-terminal domain [135].

Within murine mesenteric arteries, no difference in the abundance of K_V_1.5 was detected between males and females [136], however whole cell K_V_ currents were greater in mesenteric arteries from female mice [136] and rats [66]. Though estrus cycle regulation of vascular K_V_1 channels has yet to be investigated, Saito *et al.* (2009) observed diminished K_V_1.5 currents and protein in murine ventricular myocytes harvested from females in estrus when compared to di-estrus [45]. Further, supplemental E2 inhibited ventricular K_V_1.5 currents and protein expression in ovariectomized mice [45] and tamoxifen increased the relative abundance of K_V_1.5 [41]. Some evidence indicates that levels of progesterone seen during pregnancy can impair K_V_1.3/1.5 transcript expression in native cardiac myocytes and heterologous expression systems [46]. Within the vasculature, testosterone deprivation impairs K_V_ current and K_V_1.5 expression in aortic VSMCs [49]. Similarly, within ventricular myocytes, castration diminished and testosterone replacement enhanced K_V_1.5 expression and function [47,48]. Evidence therefore exists for estrus cycle dependent regulation of K_V_1 channels [45], with E2 impairing and testosterone promoting K_V_1 function, though this remains to be investigated within the vasculature.

## K_V_2 channels

Several studies have identified *KCNB-*encoded K_V_2.1 and K_V_2.2 in whole lysates of arteries [137,138]. Of note, K_V_2.2 is minimally expressed compared to K_V_2.1 in rat middle cerebral artery [139] and aorta [140]. As K_V_2.1 and K_V_2.2 form heterotetramers [141], the effects of K_V_2.2 can be difficult to delineate. Consequently, the current literature has focused predominantly on K_V_2.1 by comparison to K_V_2.2. K_V_2.1 contributes to K_V_ currents in rat pulmonary [142], mesenteric [143] and aortic [144,145] VSMCs. Functionally, K_V_2.1 regulates resting *V*_m_ in rat middle cerebral artery and opposes myogenic tone in a pressure-sensitive process [146] in heteromultimeric configurations of K_V_2.1/K_V_9.3 [139]. K_V_2.1 is also upregulated in response atrial natriuretic peptide- and NO-dependent signaling within the rat aorta [140].

The only study to consider sex-differences in vascular K_V_2 channels to date found that mesenteric VSMCs from female C57BL/6J mice expressed greater K_V_2.1 protein and current when compared to males [136]. Paradoxically, these findings were observed in conjunction with enhanced Ca_V_1.2 open probability, greater pressure mediated [Ca^2+^]_i_ increase and pressure induced tone within arteries from female mice [136]. To explain these contrasting findings, O’Dwyer *et al.* (2020) argue for a novel role for non-conductive K_V_2.1 channels in regulating VSMC plasmalemmal Ca_V_1.2 channel clustering and function [136]. Thus, in their model, a greater ratio of K_V_2.1:Ca_V_1.2 in arteries from female animals leads to enhanced Ca^2+^ influx and constriction [136].

Though the estrus cycle was not considered in the previous study, some evidence exists for the regulation of K_V_2 by sex hormones. Supplemental E2 diminishes both native K_V_2.1 currents in osteocytes, in a process speculated to be non-genomic [50], and over-expressed K_V_2.1 in cultured hippocampal neurons [51]. Further, ER inhibition with tamoxifen increases K_V_2.1 expression in guinea pig heart [41], ER_β_ signaling impairs K_V_2.1/2.2 currents in β-pancreatic cells [52] and GPER1 activation inhibits K_V_2.2 expression in a PKC-sensitive signaling cascade [53]. Though in murine ventricular myocytes, no estrus cycle-sensitive change in K_V_2.1 current or protein expression was observed, which calls into question whether Kv2 channels are regulated by estrous/menstrual cycle-dependent changes in E2 levels [45]. The role of progesterone in regulating K_V_2 is even less clear. Progesterone (1 µmol-L^−1^) diminished K_V_2.1 expression in murine cardiac myocytes via mPR, which reportedly underpins prolonged QT intervals and action potential duration [46]. Though progesterone (10 µmol-L^−1^) had no effect on K_V_2.1-encoded currents in HEK 293 cells [54]. The reason for this discrepancy is currently unclear, however, a serum concentration of 1 µmol-L^−1^ progesterone is not achieved during pregnancy [147]. As such, estrus cycle regulation of vascular K_V_2 channels remains speculative; however, sex differences in vascular K_V_2 expression and function is indicated and merits further investigation.

## K_V_11.1 channels

Isoforms of *KCNH2* encode K_V_11.1, commonly known as *ether-a-go-go* (ERG)-related channels, including ERG1a and ERG1b, contribute to the fast-component of the delayed rectifying current (QT interval) of the cardiac action potential [148,149]. Within the vasculature, currents sensitive to the ERG1 blocker, E-4031, were demonstrated in murine portal vein [150,151], and K_V_11 protein and transcript were identified within isolated murine aortic, carotid and femoral arterial smooth muscle cells [152], though no ERG currents were detectable. Modulation of K_V_11.1 did however alter VSMC proliferation [152]. Additionally, K_V_11.1 inhibitor dofetilide had no functional effect in quiescent vessels, including rat aorta, mesenteric and intralobar arteries [153]. The current consensus is that K_V_11.1 functionally contributes to repolarization kinetics of spontaneously contractile smooth muscle, including portal vein [62,151,152], esophageal [154], jejunum [155], myometrial [62], and bladder [156], as well as playing a role in VSMC proliferation.

No papers to date have investigated the role of K_V_11.1 within the vasculature of the female; however, a host of literature has investigated the role of sex hormones in regulating cardiac ERG channels. Ablation of E2 production by aromatase knockout in a mouse model impaired QT interval sensitivity to E-4031 [55]. Furthermore, E2 drives K_V_11.1 forward trafficking in cardiomyocytes by enhanced interaction with HSP90, thereby increasing ventricular repolarization [57]. However, the E2 receptor inhibitor tamoxifen increased K_V_11.1 protein expression [41] and physiological concentrations of E2 reduce K_V_11.1 currents in guinea pig hearts [56] and HEK cells [58]. Additionally, mutagenesis [56], pharmacological [58], and computational [59] studies indicate that E2 binds to the K_V_11.1 pore in a manner similar to K_V_11.1 inhibitor dofetilide [59].

In contrast to E2, the role of progesterone and testosterone derivatives in regulating K_V_11.1 is clearer. Physiological levels of progesterone seen during the follicular phase upregulate cardiac K_V_11.1 currents in guinea pig ventricular myocytes [60]. Though progesterone, as seen during pregnancy, impairs K_V_11.1 forward trafficking in rat neonatal cardiac myocytes [61]. Similarly, the considerable impact of K_V_11.1 blockers on myometrial contractility is lost in pregnant mice and humans as they progress through pregnancy [62,63]. This effect is associated with an increase in KCNE2 proteins as labor develops [62,63], reinforcing the crucial role for auxiliary subunit proteins in K^+^ channel regulation and as the site of hormone based regulation. Similarly to progesterone, testosterone improves native K_V_11.1 function in HEK cells [64] and rabbit ventricular myocytes in a non-genomic post-transcriptional mechanism which enhances K_V_11.1 current density and voltage sensitivity [65].

These findings implicate a cyclical regulation of E2 suppression and progesterone promotion of K_V_11.1 function across the estrus cycle, either through channel block or through auxiliary subunit regulation, respectively. As no ERG currents have been demonstrated in K_V_11.1 expressing quiescent systemic vascular beds [152], the functional consequence for sex hormones in mediating sex-differences in K_V_11.1 regulation may be slight. Though in light of the role of ERG in mediating VSMC proliferation, there is a potential role for androgenic regulation of K_V_11.1 in sex-differences in vessel size [157], angiogenesis [158], vascularization [159], and angiogenesis [160].

## BK_Ca_ channels

*KCNMA1* encodes for the pore-forming α subunit K_Ca_1.1, which in turn gives rise to homotetrameric channels within the membrane termed BK_Ca_. Unique amongst K_Ca_, BK_Ca_ are both Ca^2+^ and voltage activated [161–163]. Within the vasculature, BK_Ca_ expression is predominantly restricted to VSMCs, though some expression may be present within the endothelium [164,165]. Vascular BK_Ca_ channels primarily couple with the K_Ca_β1 auxiliary subunit, which improves [Ca^2+^]_i_ and voltage sensitivity when compared to K_Ca_1.1 alone [166–168]. In a vascular bed specific manner, inhibitors of BK_Ca_ channels increase basal and pressure-induced tone [11,169–173]. These descriptions provide only a brief insight into the role of BK_Ca_ channels within the vasculature, for more detail see [174].

BK_Ca_ channels have been identified in male and female human mesenteric [175] and coronary [176] arteries, though data was not separated dependent on sex. Functional BK_Ca_ channels have also been demonstrated in omental and myometrial arteries from women [177,178]. Within rodents, BK_Ca_-derived currents are greater within middle cerebral artery VSMCs isolated from adult female rats when compared to age-matched males, though this phenotype dissipates with age, due to a decrease in K_Ca_β1 expression [66]. Though the estrus cycle was not factored into this study, ovariectomy diminished BK_Ca_ channel expression and enhanced pressure-mediated increases in vessel tone [66] and BK_Ca_ expression and vessel function was ameliorated by supplemental E2 dietary pellets [66].

Mechanistic insight into E2-mediated changes in BK_Ca_ function indicate that E2 (0.02–5 µmol-L^−1^)-GPER1 signaling upregulated vascular BK_Ca_ currents in human [68,69] and porcine [70] coronary artery VSMCs, via PKC. Further, ER_α_-derived PI3K signaling increased BK_Ca_1.1 transcript expression in cultured human coronary and aortic VSMCs [71] and E2 (0.3 nmol-L^−1^) treatment increased BK_Ca_ currents in ovine uterine arteries via K_Ca_β1 modulation [72–74]. The importance of the discrepancies in the receptor responsible for E2-mediated increases in vascular BK_Ca_ activity is debatable, as E2 has similar affinities for both ER_α/β_ and GPER1 [179]. These data strongly support an upregulation of BK_Ca_ channels in VSMCs by E2, although it should be noted that outside VSMCs, including in cultured human endothelial cells [67], BK_Ca_ channel expression and function is negatively regulated by E2 [180].

Investigations regarding the effect of progesterone on vascular BK_Ca_ are sparse. In heterologous expression systems, progesterone (10 µmol-L^−1^) inhibits BK_Ca_ derived currents [75], in a process speculated to be due to an increase in intracellular pH, and a decrease in cytosolic Ca^2+^/cAMP. Though progesterone (0.1 µmol-L^−1^) has no effect on native BK_Ca_ currents in uterine artery VSMCs from sheep [72].

Conversely, testosterone can enhance BK_Ca_-mediated relaxations through activation of the NO-PKG cascade in porcine coronary [76,77] and rat mesenteric arteries [78]. As both serum testosterone and E2 are raised significantly during proestrus [17], current literature would indicate an increase in vascular responsiveness during proestrus. In contrast to this hypothesis, we observed no change in relaxation response to the BK_Ca_ channel activator NS11021 in mesenteric nor renal arteries harvested from female animals across the estrus cycle [15]. The reason for this discrepancy remains to be determined and requires further investigation.

## K_ATP_ channels

ATP-sensitive K^+^ channels (K_ATP_) are a member of the inwardly rectifying K^+^ channel (K_IR_) subfamily and are derived from K_IR_6.x proteins. K_ATP_ however express poor inward rectification by comparison to other K_IR_ channels [181,182]. Expression of K_IR_6.x alone does not form functional K^+^ channels, but requires the co-expression of sulphonylurea receptors (SURs) [183–187], including SUR1, SUR2A and SUR2B [188]. K_ATP_ are now recognized as intracellular sensors that convey changes in metabolic states to electrical signals across the membrane. K_ATP_ inhibitors contract coronary [189,190] and renal arteries [191] in normotensive animals, implicating K_ATP_ in the regulation of basal arterial tone. K_ATP_ is the target of myriad receptor-mediated signaling cascades, for more detail, see [174].

Functional K_ATP_ channels are an essential components of human chorionic plate, umbilical and myometrial artery reactivity [178,192–195]. K_ATP_ channels have been demonstrated in middle cerebral, meningeal, superficial temporal and cutaneous arteries within male and female humans [196–200]. Reports of sexual dimorphisms in vascular K_ATP_ function are contradicting. For example, there are no apparent sex-dependent differences in sensitivity to K_ATP_ channel modulators in rodent cerebral [201,202] nor mesenteric [203] arteries from normotensive rats, though a later study found impaired K_ATP_ activator mediated relaxation within arteries from male hypertensive rats when compared to females [203]. Similarly, inhibition of K_ATP_ had greater impact on skeletal muscle tissue oxygenation in female rats when compared to males [204], reportedly as a result of increased VSMC K_ATP_ activity in female rats [204].

As regards to the role of sex hormones in regulating K_ATP_, E2 protects against ischemic reperfusion injury within myocardium [79] and neurons [85] by enhanced mitochondrial-K_ATP_ (mKATP) and K_IR_6.2/SUR1 function, respectively. mKATP channels when activated prevent apoptosis, enhance respiration and reduce matrix Ca^2+^ accumulation [205]. E2-mediated upregulation of K_ATP_ function is reported as both a genomic and non-genomic process, for example, E2 upregulates SUR expression within myocardial and neuronal cells [79,84,85] and also gives rise to a PKA-PKC-mediated increases in K_ATP_ activity in neuronal cells [86,87]. Similarly within humans, following angioplasty, E2 protects against myocardial ischemia by increasing myocardial K_ATP_ activity and through enhanced coronary artery vasodilation [83], however, increased coronary artery vasodilation in response to E2 was insensitive to K_ATP_ inhibition [83]. In contrast to neuronal and myocardial cells, E2 rapidly inhibited K_ATP_ function within murine β-pancreatic cells via ERβ [80,81] in a cGMP-PKG-sensitive signaling cascade [82]. Consequently, the role of E2 in regulating vascular K_ATP_ expression or activity remains speculative, and may be either positive or negative.

A comprehensive study on the effect of progesterone on K_ATP_ is yet to be undertaken. Within our own study, we observed an estrus cycle-dependent shift in sensitivity to relaxations elicited by a K_ATP_ activator within mesenteric, but not renal, arteries from female rats within proestrus/estrus, when compared to arteries from female rats within diestrus/metestrus [15]. When comparing serum sex hormone concentration between these groups we observed raised E2 in the former and raised progesterone in the later [15]. Consequently, it is unclear if mesenteric vascular K_ATP_ channel function is impaired by E2 or enhanced by progesterone [15]; however, pinacidil sensitivity was comparable between arteries from females of a low-serum estradiol stage of the estrus cycle and arteries from male animals (data not published), indicative of E2 inhibition of vascular K_ATP_ function. Further, the cause of the vascular bed specific estrus cycle regulation of K_ATP_ channel function was also unclear.

Finally, conflicting reports indicate testosterone as either a K_ATP_-dependent or K_ATP_-independent vasodilator [206]. For example, testosterone mediates K_ATP_ sensitive vasorelaxation in canine coronary [88], rat aortic [90] and human corporal [91] arteries, either via release of endothelial derived relaxant factors [88,90] or via direct interaction with the channel [91]. Conversely, the K_ATP_ inhibitor Glibenclamide had no effect on testosterone-mediated relaxation in rat aorta [89]. The reason for the discrepancy between Honda *et al.* (1999) and Ding and Stallone (2001), may be accounted for by differences in rat models. Both studies employ similar concentrations of both testosterone and Glibenclamide yielding conflicting results, warranting further investigation.

## Conclusion

This brief review demonstrates a range of extravascular and vascular studies, which detail sexual dimorphisms in ion channel function. These differences are further complicated by genomic and non-genomic sex hormone regulation of ion channels. However, fluctuations in sex hormone levels across the estrus cycle is rarely taken into consideration and studies commonly favor supraphysiological concentrations of sex hormones to investigate their role. Our lack of understanding of female biology from a basic preclinical science perspective has culminated in a greater prevalence in adverse drug reactions within women when compared with age-matched men [207]. This review highlights the need to revisit basic principles of ion channels within the vasculature of the female, with keen consideration of the estrus cycle.

## Data Availability

Data sharing is not applicable to this article.
